# Supplementary material for: Incipient sympatric speciation in wild barley caused by geological-edaphic divergence
Source: Life Sci Alliance. 2020 Oct 20;3(12):e202000827. doi: 10.26508/lsa.202000827 (PMC7652381; doi:10.26508/lsa.202000827)
Supplement: Supplementary file 1 [file LSA-2020-00827_TableS1.docx]

**Supporting Information**

**Sympatric speciation in wild barley genome caused by edaphic divergence at Evolution Plateau, Israel**

Kexin Li^1,2,3#^, Xifeng Ren^1#^, Xiaoying Song^2^, Xiujuan Li^4^, Yu Zhou^1^, Eli Harev^3^, Dongfa Sun^1*^, Eviatar Nevo^3*^

Table S1 Data quantity and quality of the samples used in the present study

| **Sample** | **Raw Base(bp)** | **Clean Base(bp)** | **Effective Rate(%)** | **Error Rate(%)** | **Q20(%)** | **Q30(%)** | **GC Content(%)** |
| --- | --- | --- | --- | --- | --- | --- | --- |
| B12 | 78,057,827,700 | 77,900,763,000 | 99.8 | 0.03 | 95.09 | 90.42 | 45.62 |
| C31 | 64,755,817,800 | 64,538,023,500 | 99.66 | 0.03 | 97.81 | 95.88 | 44.77 |
| C34 | 63,368,169,600 | 63,221,981,100 | 99.77 | 0.03 | 95.35 | 91.18 | 45.04 |
| C35 | 64,677,915,300 | 64,461,175,200 | 99.66 | 0.03 | 98.02 | 96.25 | 44.99 |
| C5 | 61,742,125,800 | 61,570,516,800 | 99.72 | 0.02 | 98.09 | 96.19 | 44.94 |
| C53 | 61,660,773,600 | 61,489,095,000 | 99.72 | 0.02 | 98.2 | 96.42 | 44.95 |
| B16 | 76,024,181,100 | 75,926,804,700 | 99.87 | 0.04 | 97.97 | 96.84 | 45.43 |
| B21 | 83,777,349,300 | 83,617,733,700 | 99.81 | 0.04 | 97.72 | 96.45 | 44.97 |
| B25 | 73,933,519,800 | 73,821,401,100 | 99.85 | 0.04 | 97.93 | 96.76 | 45.73 |
| B26 | 74,201,481,300 | 74,079,169,500 | 99.84 | 0.04 | 97.81 | 96.58 | 45.5 |
| B44 | 77,536,457,100 | 77,421,842,400 | 99.85 | 0.04 | 97.85 | 96.66 | 44.86 |
| B59 | 70,909,777,800 | 70,755,576,000 | 99.78 | 0.04 | 97.85 | 96.66 | 44.89 |
| B62 | 91,552,211,400 | 91,363,778,100 | 99.79 | 0.04 | 97.75 | 96.5 | 44.82 |
| B8 | 71,815,943,100 | 71,714,767,800 | 99.86 | 0.04 | 98.03 | 96.94 | 45.22 |
| C66 | 64,777,681,800 | 64,700,175,600 | 99.88 | 0.04 | 97.22 | 95.59 | 44.77 |
| B34 | 66,568,500,600 | 66,483,422,400 | 99.87 | 0.04 | 95.77 | 93.62 | 44.81 |
| B40 | 81,728,207,700 | 81,621,224,400 | 99.87 | 0.04 | 95.77 | 93.62 | 45.17 |
| C13 | 83,619,452,400 | 83,489,502,900 | 99.84 | 0.04 | 95.53 | 93.3 | 44.77 |
| C15 | 74,436,284,700 | 74,345,485,200 | 99.88 | 0.04 | 95.57 | 93.34 | 44.66 |
| C24-1 | 30,877,506,900 | 30,852,149,700 | 99.92 | 0.03 | 97.58 | 95.21 | 45.47 |
| C24-2 | 30,942,366,000 | 30,916,603,200 | 99.92 | 0.03 | 97.71 | 95.51 | 45.5 |
| C3-1 | 32,981,792,100 | 32,940,192,900 | 99.87 | 0.03 | 97.68 | 95.39 | 45.78 |
| C3-2 | 33,042,386,400 | 32,999,337,900 | 99.87 | 0.03 | 97.8 | 95.67 | 45.81 |
| C9-1 | 35,931,279,000 | 35,894,308,200 | 99.9 | 0.03 | 97.62 | 95.28 | 46.06 |
| C9-2 | 36,039,463,500 | 36,001,311,300 | 99.89 | 0.03 | 97.74 | 95.57 | 46.09 |
| B52-1 | 44,651,765,700 | 44,546,171,400 | 99.76 | 0.04 | 97.9 | 96.75 | 45.42 |
| B52-2 | 44,624,292,900 | 44,518,057,500 | 99.76 | 0.04 | 97.87 | 96.69 | 45.41 |
| B63-1 | 40,104,417,000 | 40,061,257,500 | 99.89 | 0.04 | 97.22 | 95.69 | 45.19 |
| B63-2 | 39,789,124,500 | 39,745,557,000 | 99.89 | 0.04 | 97.14 | 95.59 | 45.2 |
| *-1and*-2are the same individual but from two different lanes | | | | | | | |
