# Supplementary material for: Incipient sympatric speciation in wild barley caused by geological-edaphic divergence
Source: Life Sci Alliance. 2020 Oct 20;3(12):e202000827. doi: 10.26508/lsa.202000827 (PMC7652381; doi:10.26508/lsa.202000827)
Supplement: Supplementary file 2 [file LSA-2020-00827_TableS2.docx]

**Supporting Information**

**Sympatric speciation in wild barley genome caused by edaphic divergence at Evolution Plateau, Israel**

Kexin Li^1,2,3#^, Xifeng Ren^1#^, Xiaoying Song^2^, Xiujuan Li^4^, Yu Zhou^1^, Eli Harev^3^, Dongfa Sun^1*^, Eviatar Nevo^3*^

Table S2 Reads mapping quality and coverage

| Sample | Clean_reads | mapped_reads | mapping_rate | Average_depth | Coverage_1x | Coverage_4x |
| --- | --- | --- | --- | --- | --- | --- |
| C5 | 410470112 | 403557735 | 98.32% | 11.5 | 90.43% | 78.61% |
| C53 | 409927300 | 403108958 | 98.34% | 11.61 | 90.42% | 79.17% |
| C31 | 430253490 | 423172922 | 98.35% | 12.8 | 90.66% | 80.44% |
| C35 | 429741168 | 422584124 | 98.33% | 12.35 | 90.78% | 80.59% |
| C24 | 411791686 | 404670838 | 98.27% | 12.61 | 90.82% | 80.67% |
| C3 | 439596872 | 431745857 | 98.21% | 13.53 | 90.77% | 80.97% |
| C9 | 479304130 | 470457358 | 98.15% | 14.55 | 91.01% | 81.58% |
| C13 | 556596686 | 545329969 | 97.98% | 18.39 | 92.56% | 85.70% |
| C15 | 495636568 | 485976246 | 98.05% | 16.62 | 92.17% | 84.53% |
| C34 | 421479874 | 412543353 | 97.88% | 13.11 | 91.42% | 81.87% |
| C66 | 431334504 | 424534247 | 98.42% | 13.83 | 91.24% | 82.19% |
| B8 | 478098452 | 470842786 | 98.48% | 15.17 | 91.18% | 82.68% |
| B25 | 492142674 | 484216193 | 98.39% | 15.59 | 91.57% | 83.12% |
| B34 | 443222816 | 434638879 | 98.06% | 14.81 | 91.96% | 83.65% |
| B16 | 506178698 | 498339687 | 98.45% | 15.99 | 91.90% | 84.09% |
| B12 | 519338420 | 508485216 | 97.91% | 15.58 | 92.41% | 84.36% |
| B44 | 516145616 | 508252583 | 98.47% | 16.25 | 92.03% | 84.41% |
| B63 | 532045430 | 522922381 | 98.29% | 17.05 | 92.09% | 84.90% |
| B62 | 609091854 | 599398611 | 98.41% | 18.83 | 92.19% | 85.59% |
| B40 | 544141496 | 532285050 | 97.82% | 17.94 | 92.56% | 85.56% |
| B21 | 557451558 | 548380136 | 98.37% | 17.33 | 92.21% | 85.15% |
| B26 | 493861130 | 485757365 | 98.36% | 15.44 | 91.88% | 83.98% |
| B52 | 593761526 | 584032561 | 98.36% | 18.14 | 91.98% | 85.19% |
| B59 | 471703840 | 464047592 | 98.38% | 15.03 | 91.60% | 83.47% |
