# Supplementary material for: Incipient sympatric speciation in wild barley caused by geological-edaphic divergence
Source: Life Sci Alliance. 2020 Oct 20;3(12):e202000827. doi: 10.26508/lsa.202000827 (PMC7652381; doi:10.26508/lsa.202000827)
Supplement: Supplementary file 3 [file LSA-2020-00827_TableS3.docx]

**Supporting Information**

**Sympatric speciation in wild barley genome caused by edaphic divergence at Evolution Plateau, Israel**

Kexin Li^1,2,3#^, Xifeng Ren^1#^, Xiaoying Song^2^, Xiujuan Li^4^, Yu Zhou^1^, Eli Harev^3^, Dongfa Sun^1*^, Eviatar Nevo^3*^

**Table S3** Distribution of the SNPs across the genome of chalk and abutting basalt populations

|  | Category | Number of SNPs | |
| --- | --- | --- | --- |
|  |  | Basalt | Chalk |
|  | Upstream | 14759 | 86147 |
| Exonic | Stop gain | 342 | 1758 |
| Exonic | Stop loss | 50 | 304 |
| Exonic | Synonymous | 8560 | 52810 |
| Exonic | Non-synonymous | 10346 | 59857 |
|  | Intronic | 44821 | 253606 |
|  | Splicing | 89 | 414 |
|  | Downstream | 15302 | 86061 |
|  | upstream/downstream | 1173 | 6283 |
|  | Intergenic | 1920192 | 12007898 |
|  | ts | 1231040 | 7717017 |
|  | tv | 784594 | 4838121 |
|  | ts/tv | 1.569 | 1.595 |
|  | Total | 2015634 | 12555138 |

Ts: transition, tv: transversion
